# Equations for Lipid Normalization of Carbon Stable Isotope Ratios in Aquatic Bird Eggs

**DOI:** 10.1371/journal.pone.0083597

**Published:** 2014-01-22

**Authors:** Kyle H. Elliott, Mikaela Davis, John E. Elliott

**Affiliations:** 1 Department of Biological Sciences, University of Manitoba, Winnipeg, Canada; 2 Department of Biological Sciences, Simon Fraser University, Burnaby, Canada; 3 Science & Technology Branch, Environment Canada, Delta, Canada; Phillip Island Nature Parks, Australia

## Abstract

Stable isotope ratios are biogeochemical tracers that can be used to determine the source of nutrients and contaminants in avian eggs. However, the interpretation of stable carbon ratios in lipid-rich eggs is complicated because ^13^C is depleted in lipids. Variation in ^13^C abundance can therefore be obscured by variation in percent lipids. Past attempts to establish an algebraic equation to correct carbon isotope ratios for lipid content in eggs have been unsuccessful, possibly because they relied partly on data from coastal or migratory species that may obtain egg lipids from different habitats than egg protein. We measured carbon, nitrogen and sulphur stable isotope ratios in 175 eggs from eight species of aquatic birds. Carbon, nitrogen and sulphur isotopes were enriched in lipid-extracted egg samples compared with non extracted egg samples. A logarithmic equation using the C∶N ratio and carbon isotope ratio from the non extracted egg tissue calculated 90% of the lipid-extracted carbon isotope ratios within ±0.5‰. Calculating separate equations for eggs laid by species in different habitats (pelagic, offshore and terrestrial-influenced) improved the fit. A logarithmic equation, rather than a linear equation as often used for muscle, was necessary to accurately correct for lipid content because the relatively high lipid content of eggs compared with muscle meant that a linear relationship did not accurately approximate the relationship between percent lipids and the C∶N ratio. Because lipid extraction alters sulphur and nitrogen isotope ratios (and cannot be corrected algebraically), we suggest that isotopic measurement on bulk tissue followed by algebraic lipid normalization of carbon stable isotope ratio is often a good solution for homogenated eggs, at least when it is not possible to complete separate chemical analyses for each isotope.

## Introduction

Stable isotope analysis is a useful technique for tracing the origin of nutrients in tissues with applications in environmental chemistry, paleoecology, migration biology and diet reconstruction [1]–[10]. Stable isotope analysis applied to egg tissue is particularly useful for understanding where resources are derived for reproduction (capital vs. income breeding) and to account for variation in toxic contamination within the egg due to diet [8]–[14].

Agencies in a number of countries systematically collect and archive bird eggs for toxicological and chemical analyses because eggs obtained early in the season can be re-laid, collection of 10–20 eggs has little impact on bird populations numbering in the thousands or millions, and the lipid-rich matrix accumulates many of the lipophilic toxins of interest [9], [15]–[21]. Archived egg specimen banks allow retrospective analysis of toxic contaminants. Stable isotope analysis helps tease apart whether changes in contamination on archived tissue occur due to changes in diet or changes in toxin abundance [17], [22]–[24].

The most common stable isotope ratios used by ecologists are those involving carbon (^13^C∶^12^C, measured relative to the PeeDee Belemnite standard and denoted δ^13^C) and nitrogen (^15^N∶^14^N, measured relative to pure air and denoted δ^15^N) ratios. Carbon isotopes can be used to identify habitat, as δ^13^C varies systematically with degree of aquatic and anthropogenic input. Nitrogen isotopes are primarily used to determine trophic level, as δ^15^N increases predictably with trophic level. Increasingly, sulfur isotope ratios (^34^S∶^32^S, measured relative to the Vienna Cañon Diablo Troilites standard and denoted δ^34^S) are also used to distinguish nutrients originating from marine environments. Stable isotope ratios change in a systematic fashion as nutrients are assimilated from an animal's food (i.e. the discrimination factor). An accurate knowledge of the discrimination factor is necessary to quantitatively predict nutrient origin [5], [25].

Discrimination factors differ among tissue types, with lipids being more depleted in ^13^C than protein because lipid biosynthesis preferentially incorporates ^12^C compared with protein [26]–[31]. In particular, isotopic fractionation during the oxidation of pyruvate to acetyl coenzyme A, the main precursor to fatty acids, preferentially incorporates ^12^C [32]. Thus, despite originating from the same resource, the stable isotope ratio of consumer tissue will be more depleted in ^13^C if the lipid content of that tissue is high.

There are two methods for correcting for lipid content [31], [33]–[36]. First, lipids can be extracted chemically prior to measurement, although lipid extraction can alter δ^15^N by washing out nitrogenous compounds. Measurement of δ^13^C in lipid-extracted tissue and δ^15^N in non-extracted tissue overcomes that issue, but doubles cost and work load. Furthermore, the lipid extraction process can be time-consuming and can involve the use of hazardous chemicals, such as chloroform. Second, provided an index of lipid content is available, isotope ratios can be corrected for lipid content analytically without extraction. Often, the ratio of total weight of carbon to total weight of nitrogen within the sample (C∶N ratio) is used as an index of lipid content because nitrogen abundance is high in proteins and low in lipids, and C∶N ratios are readily calculated from data obtained during stable isotope analysis [31], [33]–[36].

Lipid normalization models are equations that use the C∶N ratio or percent lipids to calculate the value of δ^13^C that would have been present in a tissue following lipid extraction from the value measured in non-extracted tissue. Such models are accurate in muscle tissue in a wide variety of animals [31], [35], [37], [38]. Furthermore, equations developed from the Post et al. dataset [31] accurately predicted 85% of the data points in a different dataset [35], suggesting that equations are robust across datasets.

Using lipid normalization models in eggs, however, has been more problematic (Table 1). For instance, δ^13^C in eggs collected from three species of wild birds varied widely between lipid-extracted and non-extracted tissue, and was not predicted by C∶N ratio [38], [39]. Similarly, the mean-square error for eggs was four times greater than for muscle (once three outliers and seven points with incomplete lipid extraction were excluded for muscle; no such points were apparently excluded for eggs, [38]). Waterfowl, in particular, show a different or inconsistent trend between lipid-extracted and non-extracted tissue [35], [39]. One potential reason for variation among studies is that coastal birds (e.g. eagles, waterfowl) may incorporate variable amounts of freshwater-derived rather than marine-derived lipids into their tissues, that variation may be independent of protein source, and freshwater-derived lipids are depleted in δ^13^C compared to marine-derived lipids [38], [39].

**Table 1:** Difference between lipid-extracted and non-extracted samples for bird egg tissue for carbon (Δδ^13^C), nitrogen (Δδ^15^N) and sulphur (Δδ^34^S).

Species	N	Solvent	Δδ^13^C	Δδ^15^N	Δδ^34^S	Relationship	Reference
Bald eagle	109	Diethyl ether	2.0±1.2‰	0.0±0.3‰		None	Ricca et al. 2007^1^
King eider	18	Chloroform-methanol	4.1±1.2‰	1.2±0.3‰	2.3±1.1‰	None	Oppel et al. 2010^2^
Spectacled eider	15	Chloroform-methanol	2.8±0.4‰	1.0±0.7‰		None	Oppel et al. 2010^2^
Snow goose	11	Chloroform-methanol	1.9‰	0.5‰		None	Ehrich et al. 2011
32 arctic species	1–4	Chloroform-methanol	3.3‰	0.6‰		Nonlinear	Ehrich et al. 2011
Glaucous-winged gull	19	Chloroform-methanol	4.5±0.7‰	0.5±0.5‰		Linear	Our study^2^
Ancient murrelet	6	Petroleum ether	2.0±0.3‰	0.7±0.4‰		Linear	Our study
Double-crested cormorant	10	Petroleum ether	1.3±0.3‰	0.4±0.2‰		Linear	Our study
Great blue heron	2	Petroleum ether	1.0±0.3‰	0.5±0.2‰		None	Our study
Leach's storm-petrel	68	Petroleum ether	2.3±0.4‰	0.9±0.5‰	−0.1±0.9‰	Linear	Our study
Osprey	12	Petroleum ether	1.0±0.6‰	0.8±0.2‰		None	Our study
Pelagic cormorant	26	Petroleum ether	1.8±0.5‰	0.9±0.4‰		Nonlinear	Our study
Rhinoceros auklet	51	Petroleum ether	2.2±0.2‰	1.3±0.3‰	1.6±1.7‰	Nonlinear	Our study
All species (except gulls)	175	Petroleum ether	2.0±0.6‰	0.9±0.5‰	0.5±1.5‰	Nonlinear	Our study

Uncertainty represents SD, where given in published studies. “Relationship” shows whether the relationship was reported to be non-significant (“None”), significant and linear (“Linear”) or significant and non-linear (“Non-linear”).

^1^ Compared percent lipids rather than C∶N ratio.

^2^ Analyzed whole yolk; all other studies examined egg homogenate.

Both linear and non-linear equations have been used to describe the relationship between the C∶N ratio and the effect of lipid extraction on tissue δ^13^C values. For tissues that have a relatively low percent lipids, such as muscle, a linear equation appears to hold (most studies show no or only slight non-linearity [31], [35], [38]; but see [40] that found support for a non-linear equation). However, a nonlinear relationship, whereby the effect of lipid extraction is smaller at higher percent lipid content, appears to work better for eggs, which usually have higher percent lipid content than muscles [35]. The mathematical necessity of a decelerating, non-linear equation for tissues with high percent lipids is evident in the fact that a mixture of pure fatty acids (100% lipids) would have a nitrogen content of zero and an infinite C∶N ratio. A linear model would then imply that the δ^13^C value would be depleted infinitely, when in fact δ^13^C would merely be depleted by 5–6‰.

Because of the paucity of data on the effect of lipid-extraction on the eggs of non-waterfowl [35], [38], we examined the effect of lipid extraction on the eggs of six species of marine seabirds and an aquatic raptor. Those seabirds were chosen in the 1980s as part of a toxic contaminant monitoring program in Pacific Canada. The species were chosen because they feed in nearshore, continental shelf and offshore environments, and, therefore, can be used to monitor contaminants in those habitats [9], [41]. In the context of the current study, the variation in habitat (freshwater vs. nearshore vs. offshore) allows us to examine the effect of habitat on the predictability of lipid normalization equations. By including multiple individuals per species, from seven different species, we can examine both intra-specific and inter-specific variability in the relationship between lipid-extracted and non-lipid-extracted δ^13^C relative to the ratio of bulk C∶N. In addition, we examined the effect of a second lipid extraction process on δ^13^C of an eighth seabird species. The goal of the manuscript is to develop an equation for normalizing for the effect of lipids on whole egg homogenate, to examine whether that relationship is linear or nonlinear, and to determine whether the relationship is species- or habitat-specific.

## Materials and Methods

We randomly selected archived egg contents from the Environment Canada's Specimen Bank [42]. We did not control for laying order (storm-petrels and auklets only lay a single egg), or days since lay, which can affect stable isotope ratios in eggs [43], [44]. As we collected eggs randomly during early incubation, we do not believe those issues created systematic bias. Eggs were collected from a nearshore marine seabird (double-crested cormorant *Phalacrocorax auritus*), three seabirds that forage primarily on the continental shelf during the breeding season (ancient murrelet *Synthliboramphus antiquus*, rhinoceros auklet *Cerorhinca monocerata*, pelagic cormorant *Phalacrocorax pelagicus*), one offshore-foraging marine seabird (Leach's storm-petrel *Oceanodrama leucorhoa*), a freshwater aquatic raptor (osprey *Pandion haliaetus*) and an aquatic bird that also uses the terrestrial environment (great blue heron *Ardea herodias*). For the purposes of analysis, we classified bird species into three habitat types: offshore (Leach's storm-petrel), continental shelf (rhinoceros auklet and pelagic cormorant) and those in habitats influenced by the terrestrial environment, such as lakes or estuaries (ancient murrelet, osprey, double-crested cormorant and great blue heron). Egg contents were homogenized and frozen until analysis.

After thawing, samples were freeze-dried. A small sample (1 mg) was removed, encapsulated in tin, and sent to the Stable Isotope Facility at University of California, Davis (http://stableisotopefacility.ucdavis.edu). On a separate sample, we extracted the lipids using a Soxhlet apparatus with petroleum ether as the solvent [45]. Specifically, a thimble filled with dried sample was placed in a Soxhlet extractor and washed with petroleum ether at 94°C for 8 hours. The solvent was then distilled off and the residue dried for 60 minutes in a drying oven. We chose petroleum ether rather than chloroform∶methanol as our solvent because several Canadian government institutions are considering the restrictions on the use of chloroform, and we wished our methods to be accessible to future researchers. After extraction, a small sample (1 mg) was removed, encapsulated, and sent to the same facility. Likewise, small samples (3 mg) of lipid-extracted and non-extracted tissue were sent to the Environmental Isotope Laboratory at University of Waterloo (http://www.uweilab.ca) for sulphur analysis.

Samples were analyzed for ^13^C/^12^C and ^15^N/^14^N isotopes using a PDZ Europa ANCA-GSL elemental analyzer (EA) interfaced to a PDZ Europa 20-20 isotope ratio mass spectrometer (IRMS; Sercon Ltd., Cheshire, UK). Samples were combusted at 1000°C in a reactor packed with chromium oxide and silvered copper oxide. Following combustion, oxides were removed in a reduction reactor (reduced copper at 650°C). The helium carrier then flowed through a water trap (magnesium perchlorate). N_2_ and CO_2_ were separated on a Carbosieve GC column (65°C, 65 mL/min) before entering the IRMS. Samples were analyzed for ^34^S/^32^S using a Europa Roboprep-20/20 EA-IRMS. During analysis, samples were interspersed with several replicates of at least two different laboratory standards. The final delta values were expressed relative to international standards Vienna PeeDee Belemnite, Vienna Cañon Diablo Troilite and air for carbon, sulphur and nitrogen, respectively.

To examine the effect of a different lipid extraction process on variation in δ^13^C, we also collected egg yolk from glaucous-winged gulls (*Larus glaucescens*), a nearshore marine seabird that also uses the terrestrial environment. We used chloroform∶methanol extraction to remove lipids from egg yolk. Lipid-extracted and non-extracted egg yolks were encapsulated and sent to the UC Davis Stable Isotope Laboratory for carbon and nitrogen analyses. All data for both projects are presented in Table S1.

We used R 2.14.2 for all statistical analyses. We used linear regression and non-linear regression based on previous published logarithmic models [35] to examine the relationship between Δδ^13^C (δ^13^C_lipid extracted_ - δ^13^C_non extracted_), Δδ^15^N (δ^15^N_lipid extracted_ - δ^15^N_non extracted_), Δδ^34^S (δ^34^S_lipid extracted_ – δ^34^S_non extracted_) and the C∶N ratio. Linear models are appropriate for tissues with low percent lipids because as long as percent lipids is low a linear model approximates the nonlinear function; for tissues with high percent lipids (e.g., eggs), the C∶N ratio does not change linearly with percent lipids (because the denominator, nitrogen, approaches zero) necessitating a non-linear function [35],[40]. Because models with an increasing number of parameters will necessarily increase fit while including spurious relationships (i.e., a model with 175 parameters would explain 100% of the variation in our 175-sample data set), we used Akaike's information criterion to select the most parsimonious relationship. We considered both linear and non-linear functions, and functions that included species and habitat (offshore, shelf and terrestrial-influenced), and their interactions, as co-variates. We then examined whether each of the 175 values were predicted within 0.5‰ by each model, with each model recalculated to exclude that point. Because many authors will only examine these relationships within a single species, we also calculated species-specific regressions to determine the likelihood of finding a relationship given only a single species with a more limited range of lipid content. We also correlated δ^13^Clipid-extracted and δ^13^Cnon-extracted. We removed eight values, all from the same Soxhlet run, because the C∶N ratio after lipid extraction was >5.0. All raw data are available in Table S1.

## Results

**Figure 1:**
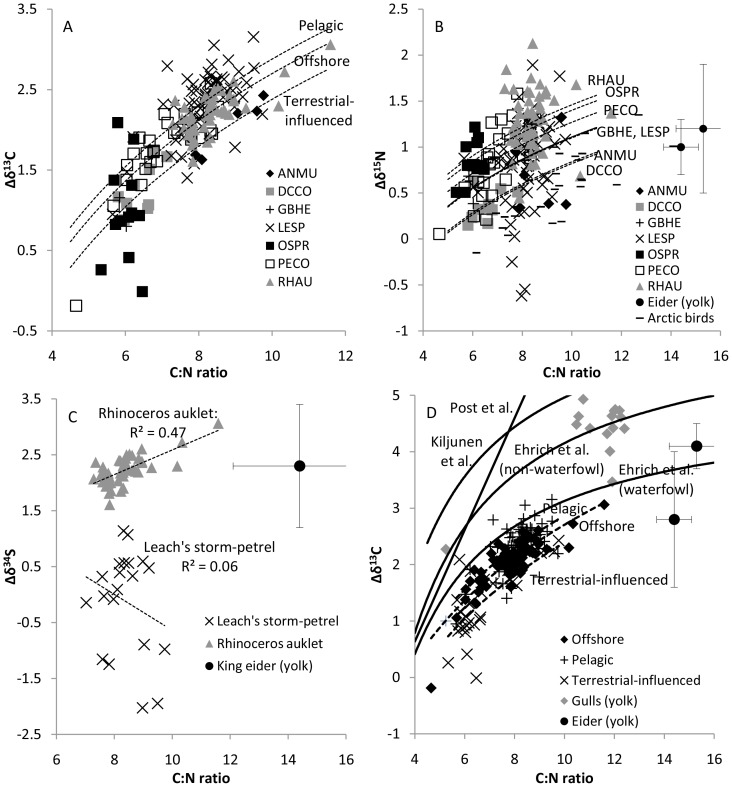
Difference between lipid-extracted and non-extracted stable isotope ratios for bird egg tissue. Specifically (A) carbon (Δδ^13^C), (B) nitrogen (Δδ^15^N) and (C) sulphur (Δδ^34^S) increases with ratio of carbon to nitrogen by weight (C∶N ratio) across seven aquatic bird species: ancient murrelet (ANMU), double-crested cormorant (DCCO), great blue heron (GBHE), Leach's storm-petrel (LESP), osprey (OSPR), pelagic cormorant (PECO) and rhinoceros auklet (RHAU). Also shown are results from studies listed in Table 1 (eider average with SD bars shown, Arctic birds) and best-fit habitat- and species-dependent regression models listed in Table 2. (D) Δδ^13^C for groups within our study compared with arithmetic lipid-correction models proposed by Post et al. [29], Ehrich et al. [33] (filled lines) and within our own study (dashed lines).

Lipid-extracted egg samples were more enriched in carbon, sulphur and nitrogen than non-extracted egg samples (Table 1). Across the entire dataset, there was strong support for a logarithmic relationship between Δδ^13^C and the C∶N ratio (Table 2, Fig. 1). Relationships between Δδ^15^N and the C∶N ratio, and Δδ^34^S and the C∶N ratio, were weaker, but still supported (Table 2). For the entire dataset of 175 eggs, 22 (12%) of the lipid-extracted egg values differed by more than 0.5‰ in δ^13^C when calculated using the linear model, 19 (10%) differed by more than 0.5‰ for the logarithmic model and 12 (7%) differed by more than 0.5‰ from the complete model with habitat and species interactions included. Nonetheless, the best-supported model included separate terms in the intercept (but not the slope) for each habitat (Table 2). In contrast, 39 (22%) of the lipid-extracted egg values differed by more than 0.5‰ compared with the linear “waterfowl” equation from Ehrich et al. (2011; their Table 2, “Linear”) and 173 (99%) of the values compared with the linear “non-waterfowl” equation. Lipid-extracted and algebraically-corrected δ^13^C values were highly correlated (R^2^ = 0.990; compared with R^2^ = 0.969 for the non-algebraically corrected values). Average repeatability (standard deviation in ‰, N = 19) for duplicates (separately homogenized) was 0.26 for δ^34^S, 0.12 for δ^13^C and 0.31 for δ^15^N.

**Table 2:** Ranking of models used to describe the difference between lipid-extracted and non-extracted bird egg tissue.

Model	ΔAIC	Equation
Δδ^13^C		
Species	102.38	
Linear	54.48	
Non-linear	38.08	−4.46±0.35+7.32±0.40 * Log (C∶N Ratio)
Linear+Species	17.16	
Linear+Habitat	11.10	
Non-linear+Species+Non-linear*Species	10.21	
Non-linear+Species	6.99	
Non-linear+Habitat+Non-linear*Habitat	3.97	
Non-linear+Habitat	0.00	−3.65±0.34+6.03±0.40 * Log (C∶N Ratio)+0.32±0.07 (If Offshore)+0.50±0.07 (If Pelagic)
Δδ^15^N		
Linear	25.97	
Non-linear	24.84	−1.47±0.49+2.72±0.55 * Log (C∶N Ratio)
Habitat	22.01	
Species	8.84	
Non-linear+Habitat	8.43	
Non-linear+Species+Nonlinear*Species	7.03	
Linear+Species	1.53	
Non-linear+Species	0.00	−1.66±0.74+2.50±0.76 * Log (C∶N Ratio)−0.02±0.24 (If double-crested cormorant)+0.27±0.37 (If great blue heron)+0.26±0.18 (If Leach's storm-petrel)+0.56±0.24 (If osprey)+0.43±0.20 (If pelagic cormorant)+0.62±0.18 (If rhinoceros auklet)
Δδ^34^S		
Non-linear	8.57	
Linear^5^	8.50	4.69±3.51+−0.50±0.43 (C∶N Ratio)
Non-linear+Species+Species*Non-linear	1.61	
Linear+Species+Species*Linear	1.59	
Species	0.58	
Non-linear+Species	0.02	
Linear+Species	0.00	0.93±3.23+−0.12±0.38 * (C∶N Ratio)+1.68±0.50 (If rhinoceros auklet)

Equations are shown for the most parsimonious complete models and most-parsimonious species- and habitat-independent models. Habitat classifications were “terrestrially-influenced” (default), “offshore” (continental shelf) or “pelagic” (beyond the shelf).

There was a statistically-significant relationship between Δδ^13^C and the C∶N ratio for all of the species with sample sizes greater than two (ancient murrelet: t_5_ = 4.34, P = 0.007; double-crested cormorant: t_8_ = 3.73, P = 0.006; Leach's storm-petrel: t_66_ = 6.43, P<0.0001; pelagic cormorant: t_24_ = 7.32, P<0.0001; rhinoceros auklet: t_49_ = 6.56, P<0.0001; gull yolk: t_17_ = 4.44, P = 0.0004), except ospreys (t_10_ = −0.13, P = 0.90).

## Discussion

Lipid extraction enriched egg δ^13^C values by ∼2‰, δ^15^N values by ∼1‰ and δ^34^S by ∼0.5‰. The degree of enrichment in egg δ^13^C values (Δ^13^C) was correlated with the percent lipids, as inferred by the C∶N ratio. Indeed, across the entire dataset, and within all marine species, the C∶N ratio strongly predicted Δ^13^C. As more than 90% of the samples were estimated within ±0.5‰ by the best-fit algebraic equation (logarithm model), our algebraic equation is a robust method of calculating lipid-extracted δ^13^C values given δ^13^C values measured on non-extracted egg tissue from marine birds.

Our models for egg tissue were as accurate as those for muscle tissue [31], [35]. In contrast, most past attempts at providing a method for calculating lipid-extracted values for egg tissues have been unsuccessful (Table 1). Those methods focused on migratory or coastal species, such as bald eagles, eiders, snow geese and other waterfowl (Table 1). For migratory birds—at least those that are capital breeders—egg lipids can be derived from energy reserves obtained on non-breeding grounds with different δ^13^C signatures; egg protein, in contrast, may be derived from the breeding grounds [35], [39], [46]. Differences in δ^13^C may be particularly large for coastal birds switching between freshwater and marine prey bases [17], or for other species showing dietary switches during egg-laying [47], [48], even if they are non-migratory. For instance, the ospreys whose eggs were included in this study were tracked via satellites to wintering grounds in both marine and freshwater environments [49]. If a portion of their egg lipid δ^13^C values were derived from wintering grounds and their protein δ^13^C values were mainly derived from breeding grounds, that would explain why the lipid-extracted and non-extracted samples were consistently different [35], [39]. We therefore concur with Oppel et al. [39] that algebraic correction for the effect of lipids on δ^13^C in eggs is accurate for birds relying on resources acquired concurrently with reproduction for egg synthesis, such as income breeders, but not for coastal or migratory birds that may bring lipid reserves from a habitat with a different δ^13^C value. Specifically, algebraic corrections are applicable where lipids and proteins would be expected to be acquired in isotopically similar landscapes, with capital and income breeding being extremes of a continuum with actual reproductive strategies ranging from high to low reliance on stored nutrients [50], [51]. It is also important to emphasize that our results were primarily for egg homogenate, which has a higher proportion of protein (mostly albumin) than yolk. Arithmetic correction for yolk may be more problematic because of the lower proportion of protein in yolk and may account for problems encountered by previous authors working with yolk [39].

Stage of embryonic development also may have impacted the relationship between C∶N ratio and Δδ^13^C. As the embryo develops, there may be differential uptake of varying nutrients and, therefore, variation in ^13^CO_2_ expiration. For instance, chicken yolk δ^15^N was depleted by 1.0‰ after 15 days of development and chicken albumin δ^13^C was depleted by 0.2‰ after 3 days of development [43]. In principle, if ^13^C was excreted differentially to ^12^C in yolk relative to plasma, that could increase the variance in Δδ^13^C relative to lipid content. As we did not know the age of the embryo, this issue could play a role in our dataset, although collectors attempted to obtain eggs shortly after laying.

At the early stages of development (prior to significant growth of the embryo), the egg interior primarily consists of the cysteine-rich protein albumin (egg white) and lipid-rich yolk. Many authors have separated the two components and analyzed stable isotopes separately on each component [39], [43]. As we were interested in establishing lipid normalization equations for retrospective analyses of specimen banks for toxicological assays, where specimens have usually been homogenized, we did not examine each component separately, except for the gull eggs. Interestingly, our lipid-extracted C∶N ratio, 4.33 (SD = 0.50), is almost identical to that of egg albumin (4.28 based on the albumin amino-acid sequence), suggesting following lipid extraction on egg homogenate we were left almost entirely with albumin or with a matrix that was very similar in makeup. In particular, simple statements that pure protein should have a C∶N ratio of ∼3.0 [30] seem erroneous as the C∶N ratio will depend on the particular amino acid composition of the protein.

### The influence of habitat and breeding strategy on Δδ^13^C

Pelagic foragers (Leach's storm-petrels) had higher Δδ^13^C than nearshore foragers (rhinoceros auklets and pelagic cormorants), which had higher Δδ^13^C than birds foraging in terrestrial-influenced habitats (ancient murrelets, double-crested cormorants, great blue herons and ospreys). Ancient murrelets may forage farther offshore than pelagic cormorants, but for the current classification scheme they appeared to group with those species feeding in terrestrial-influenced habitats. Perhaps there is a larger difference in the carbon isoscape in winter (where lipids were derived) relative to breeding (where non-lipids were derived) habitat for birds feeding offshore than for those feeding in the terrestrial environment. Alternatively, within the capital-income breeding strategy continuum, Leach's storm-petrels may rely more on endogenous stores for egg production and the terrestrial-influenced species may rely more on exogenous stores for egg production [48], [50]–[52]. Ehrich et al. [35] found higher Δδ^13^C for terrestrial birds (along with one seabird and several shorebirds) than waterfowl. Either variation in habitat or breeding strategy (endogenous or capital vs. exogenous or income) may explain why Δδ^13^C differed between terrestrial birds and waterfowl in that study.

### The effect of solvent

The solvent used during lipid extraction is known to effect measurement of δ^13^C and δ^15^N, at least in fish and invertebrates [45], [52]. The ideal solvent would extract all lipids, and only lipids, but in practice both lipids and non-lipids vary in polarity; non-polar lipids, such as triglycerides, are more soluble in non-polar solvents (e.g. petroleum ether, polarity index ∼0.1) and polar lipids, such as phospholipids, are more soluble in polar solvents (e.g. 2∶1 chloroform∶methanol, polarity index ∼4.4). Thus, no solvent will extract all lipids and all solvents will extract some non-lipids, such as hydrophobic amino acid or carbohydrate derivatives. In one study, δ^13^C in lipid-extracted tissue varied among three different solvents, with chloroform-methanol (Bligh-Dyer method, [54]) causing systematic errors on one type of invertebrate tissue [45]. In contrast, another study on fish tissue found that chloroform-methanol extraction was somewhat more effective than other techniques [53].

We believe a solvent effect is present for bird eggs. The arithmetic correction for Δδ^13^C proposed by Ehrich et al. [35] is quite effective for non-waterfowl (gulls in our study) and waterfowl [39] in other studies that used chloroform∶methanol as the solvent (Fig. 1d). The Ehrich et al. [36] equation calculates higher Δδ^13^C values than our equations, which would be consistent with chloroform-methanol being a stronger solvent (extracting a greater overall portion of lipids, but also extracting some non-lipids). Furthermore, pure gull albumin had a C∶N ratio of 3.72±0.06 and lipid-extracted whole egg had a C∶N ratio of 3.72±0.04 in the gull dataset using the chloroform-methanol extraction process, whereas lipid-extracted whole egg had a C∶N ratio of 4.33±0.50 in the seabird dataset using the petroleum ether extraction process. Thus, we believe that chloroform-methanol extractions remove more, or at least different, lipids (and other molecules) than petroleum ether, and therefore cause a larger Δδ^13^C.

### Why use lipid normalization equations?

Our lipid normalization equations will be of use in accounting for the effect of lipids in toxicological studies of purely marine birds, although we urge the establishment of separate equations for each species. There are many reasons why lipid extraction is sub-optimal: chloroform is not allowed at many laboratories for health reasons, the Soxhlet apparatus used in the current studies cost over $10 000, the apparatus created a bottleneck of only eight samples processed each day, δ^13^C (and δ^15^N/δ^34^S) will vary among extraction techniques and, finally, to avoid overestimation of δ^15^N or δ^34^S (which cannot be corrected algebraically) it would be necessary to complete two different analyses, doubling expenses. Lastly, lipid extraction removes at least some amino acids, which can insert some (unwanted) variability into diet estimation. For studies of nutrient allocation or prey use, very accurate and unbiased measurements of δ^13^C may be necessary, but for toxicological studies that primarily use stable isotope ratios as correlative, predictive variables, the algebraic approach may be sufficient; across all species, lipid-extracted and algebraically-corrected values were highly correlated (R^2^ = 0.99) so that in correlative studies essentially no information would be lost by using the algebraic approach. Furthermore, where the algebraic equations do not provide an accurate index of lipid extracted δ^13^C because lipids and proteins are derived from different environments, the algebraic equations will give the value more associated with lipophilic contaminants; the stable isotope ratios measured on lipid-extracted tissue (albumin) will not reflect the diet associated with the prey from which the lipophilic contaminants are derived. We, therefore, urge the use of algebraic corrections, rather than lipid extraction, in toxicological studies.

### What equation to use?

The most-widely used equation is the Post et al. [31] equation, which is accurate for muscle from many different organisms [31], [35]. Muscle is relatively low in lipids, so the non-linearity apparent in our dataset is not a significant issue (i.e. the “Post” equation is similar to the “Ehrich” equation at low C∶N ratio, Fig. 1d). Because egg tissue can have much higher levels of lipids, a non-linear equation is necessary (Fig. 1a,d, Table 2). To estimate lipid-extracted egg tissue values (using a petroleum ether extraction process), we therefore suggest using the equation
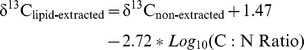
which can be increased or decreased depending on the habitat of the study species (following Table 1). The C∶N Ratio is the ratio of the weight of carbon in the sample to the weight of nitrogen in the sample, often reported in µg. The equation is not species-specific, as we did not find that species was an important term in our models.

## Supporting Information

Table S1 **Stable isotope data analyzed in the manuscript.**
(XLSX)
