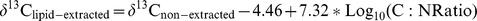# Correction: Equations for Lipid Normalization of Carbon Stable Isotope Ratios in Aquatic Bird Eggs

**DOI:** 10.1371/journal.pone.0096587

**Published:** 2014-04-24

**Authors:** The PLOS ONE Staff

In the Discussion section, there is an error in the equation for calculating d13C in non-extracted egg tissue, listed in the section titled “What equation to use?” In the equation, the d15N coefficients are switched for the d13C coefficients. Please view the complete, correct equation here: